# Postoperative pneumonia in patients with non-small cell lung cancer undergoing thoracoscopic surgery: What should we care about?

**DOI:** 10.3389/fonc.2025.1564042

**Published:** 2025-06-30

**Authors:** Jinzhi You, Dandan Chen, Xiang Liu, Hailing Zhang, Zhongfeng Zheng

**Affiliations:** ^1^ Department of Thoracic Surgery, The Affiliated Suqian Hospital of Xuzhou Medical University, Suqian, Jiangsu, China; ^2^ Department of Gastroenterology, The Affiliated Suqian First People’s Hospital of Nanjing Medical University, Suqian, Jiangsu, China

**Keywords:** pneumonia, lung cancer, surgery, treatment, care, clinical

## Abstract

**Background:**

The prevention of postoperative pneumonia in patients with non-small cell lung cancer (NSCLC) undergoing thoracoscopic surgery holds significant clinical importance. This study aimed to evaluate the status quo and influencing factors of postoperative pneumonia in patients with NSCLC.

**Methods:**

Patients with NSCLC undergoing thoracoscopic surgery at our hospital from January 2023 to October 2024 were included. The characteristics of patients with and without postoperative pneumonia were analyzed. A logistic regression model was employed to analyze the influencing factors of postoperative pneumonia, and a corresponding predictive model was constructed. The predictive performance of the model was evaluated using receiver operating characteristic (ROC) curve analysis.

**Results:**

A total of 226 patients with NSCLC were included, the incidence of postoperative pneumonia in patients with NSCLC was 31.86%. Correlation analyses showed that age(r=0.570), scope of the surgical procedure(r=0.618), COPD(r=0.562), history of smoking(r=0.516) and hypoproteinemia(r=0.587) were associated with the occurrence of postoperative pneumonia. Logistic regression analysis revealed that age (OR=2.146, 95%CI: 1.439~3.045), scope of the surgical procedure (OR=3.009, 95%CI: 2.813~3.543), COPD (OR=2.312, 95%CI: 1.605~3.008), history of smoking (OR=2.445, 95%CI: 2.117~2.821) and hypoproteinemia(OR=1.997, 95%CI: 1.533~2.580) were the independent influencing factors of postoperative pneumonia in patient with NSCLC. The area under the ROC curve (AUC) was 0.830.

**Conclusion:**

The incidence of postoperative pneumonia in patients with NSCLC is relatively high and is influenced by a multitude of factors. The postoperative pneumonia risk prediction model developed in this study has demonstrated promising predictive performance. However, given the single-center design and limited sample size, additional clinical validation is necessary to confirm its practical applicability and reliability in real-world clinical settings.

## Introduction

Lung cancer is a malignant tumor originating from the lung, trachea, or bronchus. Its cells of origin include bronchial glands, alveolar epithelial cells, and bronchial mucosal epithelial cells, with the majority of lung cancers arising from the bronchial mucosal epithelium (1, 2). There were approximately 2.21 million new cases of lung cancer worldwide, with an age-standardized incidence rate of 22.4 per 100,000 (3); meanwhile, the number of lung cancer deaths was about 1.8 million, with an age-standardized mortality rate of 18.0 per 100,000 (4). These figures indicate that lung cancer poses a significant challenge to global public health due to its high incidence and mortality rates. The pathogenesis of lung cancer has not yet been fully elucidated, but it is known that various factors may be associated with the development of lung cancer, including occupational exposure, air pollution, genetic factors, and ionizing radiation (5, 6). Among the different types of lung cancer, non-small cell lung cancer (NSCLC) is the most common, accounting for about 80% of all lung cancer cases and having a relatively high mortality rate (7, 8). The treatment options for NSCLC are diverse, encompassing surgery, radiochemotherapy, targeted therapy, and immunotherapy, with surgery being the primary method for treating NSCLC and capable of achieving a cure (9). Thoracoscopic surgery has emerged as an important minimally invasive surgical approach in the treatment of NSCLC (10). This surgical technique can effectively resect the affected tissues in patients with NSCLC, and with the continuous advancement and wider application of the technology, it has shown potential in reducing the mortality rate of NSCLC patients (11). However, it should be noted that as a form of invasive treatment, minimally invasive surgery may lead to postoperative pulmonary complications, which can affect the treatment outcomes and result in an unfavorable prognosis (12). Therefore, when implementing minimally invasive surgery for the treatment of NSCLC, it is essential to take into account the overall condition of the patient and adopt appropriate preventive and management measures to reduce the risk of postoperative complications, thereby improving the treatment outcomes and quality of life for patients (13).

Existing literature reports a wide variability in postoperative pulmonary complication rates following thoracoscopic lung cancer resection, with reported incidences spanning from 12% to 52.85%. Among these complications, postoperative pneumonia has been consistently identified as a critical prognostic determinant associated with adverse clinical outcomes. Despite its clinical significance, the epidemiological characteristics and multifactorial etiology of pneumonia following lung cancer surgery remain insufficiently characterized. This knowledge gap underscores the urgent need to establish reliable methods for identifying high-risk patients and developing accurate predictive tools to guide perioperative management strategies.

To address these clinical challenges, the present study was designed with two primary objectives: (1) to systematically evaluate the independent risk factors for postoperative pneumonia in patients undergoing thoracoscopic resection for NSCLC, and (2) to construct and validate a multivariate risk prediction model. The development of such a predictive instrument would provide clinicians with evidence-based decision support for targeted prevention strategies and personalized patient management in NSCLC surgical care.

## Methods

The present investigation was conducted as a retrospective cohort study. The study protocol underwent rigorous scrutiny and was subsequently granted approval by the hospital’s ethics committee, with the assigned approval number 2023045. In adherence to ethical standards, written informed consent was procured from each participant included in the study.

This study included patients with NSCLC who underwent thoracoscopic surgery at our hospital from January 2023 to October 2024 as the study population. The inclusion criteria were as follows: patients aged 18 years or older; diagnosed according to the diagnostic criteria for NSCLC, and postoperative pathological examination confirmed as non-small cell lung cancer; underwent thoracoscopic surgery, and no distant metastasis was found in preoperative examinations. In addition, patients needed to fully understand and voluntarily participate in this study. The exclusion criteria included: patients with other malignant tumors; patients with infectious diseases such as pneumonia and tuberculosis before surgery; and patients who were unwilling to participate in this study.

In our study, we implemented a comprehensive set of perioperative management protocols to minimize the risk of postoperative pneumonia in patients with NSCLC undergoing thoracoscopic surgery. To prevent pneumonia, we employed a multifaceted approach. Prophylactic antibiotics were administered within 1 hour before the surgical incision, with a typical regimen of a broad-spectrum cephalosporin combined with an aminoglycoside to cover a wide range of potential pathogens. This antibiotic therapy was continued for 24–48 hours postoperatively. Additionally, all patients were educated on the use of incentive spirometry during the preoperative visit and were encouraged to use it every 2 hours while awake, starting within 2 hours postoperatively, to promote lung expansion and reduce the risk of atelectasis. Early mobilization was also a key component of our strategy, with patients encouraged to mobilize within 6 hours postoperatively, assisted by nursing staff to ensure safety and facilitate improved respiratory function. Regarding anesthesia and ventilator strategies, general anesthesia was induced with propofol and fentanyl, followed by maintenance with sevoflurane or desflurane. In some cases, regional anesthesia techniques such as thoracic epidural anesthesia were used in conjunction with general anesthesia to enhance postoperative pain control and facilitate early mobilization. The surgeries performed included lobectomy and pneumonectomy utilizing video-assisted thoracoscopic surgery (VATS). During surgery, a protective ventilation strategy was employed, utilizing low tidal volumes (6–8 mL/kg ideal body weight) and positive end-expiratory pressure (PEEP) of 5–8 cm H_2_O to minimize lung injury. Permissive hypercapnia was allowed in cases of prolonged surgery or compromised lung function to maintain adequate oxygenation while reducing the risk of ventilator-induced lung injury. Periodic recruitment maneuvers were also performed to reopen collapsed alveoli and improve oxygenation, particularly in patients with pre-existing lung disease.

In this study, postoperative pneumonia was defined as pneumonia that newly occurred within 30 days after surgery in surgical patients, including cases that developed after discharge but within the 30-day postoperative period (14). The diagnosis of postoperative pneumonia required the fulfillment of the following criteria (15): Regarding radiological examination, at least two chest X-ray examinations had to be conducted (one chest X-ray examination was acceptable for patients without underlying cardiopulmonary diseases), revealing new or progressively developing and persistent pulmonary infiltrates, consolidation, or cavitation. In terms of clinical symptoms, at least one of the following had to be present: fever (temperature > 38°C) without other identifiable causes, peripheral blood leukocyte count> 12×10^9^/L or < 4×10^9^/L, or altered mental status in elderly patients aged ≥ 70 years without other identifiable causes. Regarding respiratory symptoms and signs, at least two of the following had to be observed: new onset of purulent sputum or change in sputum characteristics, increased respiratory secretions or increased suctioning requirements, new onset of cough or dyspnea or increased respiratory rate or exacerbation of pre-existing symptoms, pulmonary rales or bronchial breath sounds, or worsening of gas exchange requiring increased oxygen demand or mechanical ventilation support. These criteria, integrating radiological examination, clinical symptoms, and respiratory signs, were designed to ensure the accurate diagnosis of postoperative pneumonia.

We collected following data from the medical records of patients with NSCLC: age, gender, body mass index (BMI), pathological type, clinical staging, scope of the surgical procedure, diabetes mellitus, chronic obstructive pulmonary disease (COPD), hypertension, history of smoking, postoperative hypoproteinemia.

In this study, statistical analysis was conducted using SPSS 25.0 software. For normally distributed continuous data, results were presented as mean ± standard deviation, and comparisons between groups were made using independent samples t-tests. Non-normally distributed continuous data were expressed as median (interquartile range), with group comparisons performed using the Wilcoxon rank-sum test. Categorical data were displayed as frequency (percentage), and group comparisons were carried out using the χ² test. Pearson or spearman correlation analyses were conducted to evaluate the correlations of postoperative pneumonia with characteristics of patient with NSCLC. Furthermore, logistic regression analysis was employed in this study to investigate the risk factors influencing postoperative pulmonary pneumonia in patients with NSCLC after thoracoscopic surgery. Variables with a P-value of less than 0.10 in univariate analysis were included in the multivariate analysis model. we used a stepwise selection method (backward elimination) to identify the most significant predictors. The stepwise procedure began with the full model containing all candidate variables and iteratively removed variables with the highest p-values until all remaining variables had p-values less than 0.05. This method ensured that the final model included only the most relevant predictors while maintaining statistical rigor. Based on the variables ultimately incorporated into the multivariate model, a risk prediction model and a nomogram were constructed. To assess the robustness and generalizability of our predictive model within our study population, we performed internal validation using bootstrapping techniques. Bootstrapping is a resampling method that involves repeatedly sampling from the original dataset with replacement to create multiple bootstrap samples. This approach helps to estimate the model’s performance in a manner that simulates external validation. We generated 1,000 bootstrap samples from our original dataset and refitted the logistic regression model to each bootstrap sample. For each refitted model, we calculated the area under the receiver operating characteristic curve (AUC) to assess the model’s discriminatory ability. In this study, a P-value of less than 0.05 was considered statistically significant in this study.

## Results

A total of 226 patients with NSCLC were included in this study, of whom 72 patients had the postoperative pneumonia, the incidence of postoperative pneumonia in patients with NSCLC was 31.86%. The average duration of the surgical cases under anesthesia was 146.83 ± 22.06 minutes. As revealed in **Table 1**, there were statistical differences in the incidence of postoperative pneumonia among patients with different age, scope of the surgical procedure, COPD, history of smoking and hypoproteinemia (all p<0.05). There were no statistical differences in the gender, BMI, pathological type, clinical staging, diabetes mellitus, hypertension between patients with and without postoperative pneumonia (all p>0.05).

**Table 1:** The characteristics of included patients with non-small cell lung cancer (n=226).

Variables	Postoperative pneumonia group (n=72)	No postoperative pneumonia group (n=154)	t/χ^2^	p
Age(y)	63.21 ± 7.44	58.08 ± 8.10	8.121	0.019
Male/female	34/38	71/83	1.204	0.112
BMI (kg/m^2^)	22.64 ± 2.01	23.14 ± 2.15	3.890	0.077
Pathological type			1.438	0.095
Squamous cell carcinoma	13(18.06%)	26(16.88%)		
Adenocarcinoma	59(81.94%)	128(83.12%)		
Clinical staging			1.662	0.084
Stage I	56(77.78%)	121(78.57%)		
Stage II	10(13.89%)	25(16.23%)		
Stage III	6(8.33%)	8(5.19%)		
Scope of the surgical procedure			1.984	0.014
Partial resection	20(27.78%)	94(61.04%)		
Lobectomy	52(72.22%)	60(38.96%)		
Diabetes mellitus	11(15.28%)	19(12.34%)	1.759	0.127
COPD	28(38.89%)	21(13.64%)	1.533	0.041
Hypertension	17(23.61%)	30(19.48%)	1.696	0.105
History of smoking	29(40.28%)	20(12.99%)	1.277	0.010
Postoperative hypoproteinemia	18(25.00%)	12(7.79%)	1.852	0.002

BMI, body mass index; COPD, chronic obstructive pulmonary disease.

As indicated in **Table 2**, correlation analyses showed that age(r=0.570), scope of the surgical procedure(r=0.618), COPD(r=0.562), history of smoking(r=0.516) and hypoproteinemia(r=0.587) were associated with the occurrence of postoperative pneumonia in patient with NSCLC (all p<0.05).

**Table 2:** Correlation analysis of postoperative pneumonia with characteristics of patient with non-small cell lung cancer.

Variables	r	p
Age(y)	0.570	0.011
Gender	0.201	0.128
BMI (kg/m^2^)	0.137	0.114
Pathological type	0.188	0.076
Clinical staging	0.204	0.077
Scope of the surgical procedure	0.618	0.001
Diabetes mellitus	0.154	0.092
COPD	0.562	0.013
Hypertension	0.178	0.212
History of smoking	0.516	0.040
Postoperative hypoproteinemia	0.587	0.026

BMI, body mass index; COPD, chronic obstructive pulmonary disease.

The variable assignments of multivariate logistic regression on the influencing factors of postoperative pneumonia in patient with NSCLC are presented in **Table 3**. As indicated in **Table 4**, logistic regression analysis revealed that age (OR=2.146, 95%CI: 1.439~3.045), scope of the surgical procedure (OR=3.009, 95%CI: 2.813~3.543), COPD (OR=2.312, 95%CI: 1.605~3.008), history of smoking (OR=2.445, 95%CI: 2.117~2.821) and hypoproteinemia(OR=1.997, 95%CI: 1.533~2.580) were the independent influencing factors of postoperative pneumonia in patient with NSCLC(all p<0.05).

**Table 3:** The variable assignment of multivariate logistic regression on the influencing factors of postoperative pneumonia in patient with non-small cell lung cancer.

Factors	Variables	Assignment
Postoperative pneumonia	Y	Yes=1, no=2
Age(y)	X_1_	≥60 = 1, <60 = 2
Scope of the surgical procedure	X_2_	Lobectomy=1, partial resection =2
COPD	X_3_	Yes=1, no=2
History of smoking	X_4_	Yes=1, no=2
Postoperative hypoproteinemia	X5	Yes=1, no=2

COPD, chronic obstructive pulmonary disease.

**Table 4:** Logistic regression analysis on the influencing factors of postoperative pneumonia in patient with non-small cell lung cancer.

Variables	β	Wald	OR	95%CI	p
Age(y)	0.256	0.116	2.146	1.439~3.045	0.013
Scope of the surgical procedure	0.302	0.125	3.009	2.813~3.543	0.001
COPD	0.138	0.122	2.312	1.605~3.008	0.014
History of smoking	0.366	0.205	2.445	2.117~2.821	0.009
Postoperative hypoproteinemia	0.218	0.108	1.997	1.533~2.580	0.037

COPD, chronic obstructive pulmonary disease.

**Figure 1:**
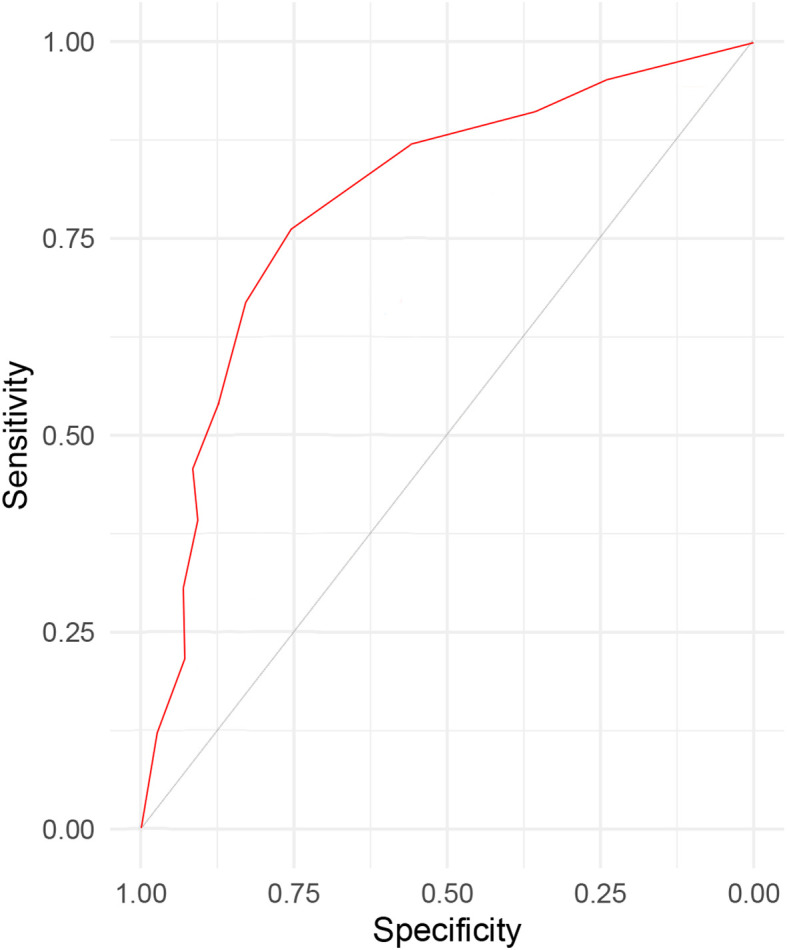
The ROC curve for the sensitivity and specificity of prediction model for evaluating postoperative pneumonia in patient with non-small cell lung cancer.

As shown in **Table 5**, the scoring criteria for the predictive model of postoperative pneumonia in patients with NSCLC are provided in detail. By utilizing the receiver operating characteristic (ROC) curve (**Figure 1**) and the scoring system of the predictive model, we calculated the sensitivity and specificity of the model at various score thresholds and determined the Youden index, which is defined as sensitivity plus specificity minus 1, as presented in **Table 6**. The analysis revealed that the Youden index was maximized when the total score was between 5.5 and 6.5. Consequently, a score of 6 was selected as the cutoff value for the model. Patients with a score higher than 6 are at a significantly increased risk of developing postoperative pneumonia. At this threshold, the model demonstrated robust sensitivity and specificity. The area under the ROC curve, along with its 95% confidence interval (CI), was found to be 0.830 (0.786, 0.882), indicating that the model has good discriminative ability and is effective in distinguishing the risk of influencing factors for postoperative pneumonia in patients with NSCLC.

**Table 5:** Logistic regression model scoring criteria for assessing postoperative pneumonia in patient with non-small cell lung cancer.

Variables	Scores
Age≥60y	2
Lobectomy	2
COPD	2
History of smoking	2
Postoperative hypoproteinemia	2

COPD, chronic obstructive pulmonary disease.

**Table 6:** Sensitivity and specificity of the prediction model at various cuff pressure thresholds.

Total score	Sensitivity	Specificity	Yorden index
-1.0	1.000	0.000	0.000
1.0	1.000	0.122	0.122
1.5	0.977	0.218	0.195
2.5	0.920	0.388	0.308
3.5	0.847	0.542	0.389
4.5	0.819	0.751	0.570
5.5	0.679	0.896	0.575
6.5	0.690	0.902	0.592
7.5	0.223	0.910	0.133
8.5	0.128	0.965	0.093
9.5	0.102	0.951	0.053
10.5	0.031	1.000	0.031
11	0.000	1.000	0.000

## Discussion

Thoracoscopic surgery, as a minimally invasive surgical approach, has been widely applied in the treatment of NSCLC. This surgical method is capable of precisely resecting the affected tissues in patients, and due to its relatively small incision size, patients tend to recover more rapidly postoperatively. This not only alleviates the patients’ suffering to a certain extent but also shortens their hospital stay (16). However, it should not be overlooked that the probability of pulmonary complications occurring in NSCLC patients after thoracoscopic surgery is relatively high, posing potential risks to the patients’ postoperative recovery (17, 18). In this study, the incidence of postoperative pneumonia is found to be 31.86%. This rate is comparable to those reported in prior relevant studies (19, 20), thereby lending further support to the clinical observation that a high incidence of pneumonia is common following thoracoscopic surgery. We have found that age, scope of the surgical procedure, COPD, history of smoking and hypoproteinemia are the independent influencing factors of postoperative pneumonia in patient with NSCLC. This finding indicates that in clinical practice, the risk management and implementation of preventive measures for pneumonia in NSCLC patients after surgery are of paramount importance. Medical staff need to enhance the monitoring of patients’ pulmonary conditions postoperatively, promptly detect and manage any potential pneumonia symptoms, and also explore and apply effective preventive strategies to reduce the incidence of pneumonia, improve the patients’ postoperative quality of life, and ensure the overall effectiveness of the surgical treatment.

However, it is essential to objectively acknowledge several limitations inherent in this study. First, the study employed a single-center research design with a relatively limited sample size. This approach may introduce biases related to demographic characteristics and geographical distribution. As a result, the generalizability of the research findings may be constrained, potentially limiting their representativeness of a broader population. Secondly, in our study, we have endeavored to comprehensively address the potential influence of institutional and systemic factors on the incidence of postoperative pneumonia. Although our primary focus was on patient-specific and surgical factors, we recognize that broader institutional and systemic issues, such as the impact of the COVID-19 pandemic and staffing challenges, may have contributed to the observed pneumonia rates. During the study period, our institution experienced several disruptions attributable to the COVID-19 pandemic. These included alterations in patient flow and resource allocation, which may have indirectly influenced the quality and consistency of postoperative care. In addition, specific data regarding the number of cases potentially related to aspiration pneumonia, as well as the number of patients with preoperative or postoperative dysphagia, were not systematically collected during the study period. We recognize the significance of these variables in elucidating the etiology of postoperative pneumonia and the potential contribution of dysphagia to aspiration events. Thirdly, we recognize that the absence of certain variables, such as forced expiratory volume in one second (FEV1) and diffusing capacity for carbon monoxide (DLCO), constitutes a limitation of our study. These pulmonary function parameters are established as significant predictors of postoperative outcomes, including the risk of pneumonia. Their omission may affect the comprehensiveness of our analysis and the interpretation of our findings. Finally, while the model developed in this study exhibited satisfactory statistical accuracy and predictive efficacy, these initial results require stringent validation via future prospective studies involving large-scale samples. Such validation is essential to ensure the model’s reliability and effectiveness in clinical settings, thereby providing a solid scientific foundation for the treatment and care of patients with NSCLC.

Age has been consistently identified as a significant risk factor for postoperative pneumonia in patients with NSCLC. Older patients are more likely to have comorbidities and reduced physiological reserves, which can impair their ability to clear respiratory secretions and increase the risk of infection. The incidence of postoperative pneumonia was found to be higher in older patients, although the specific age threshold varied across studies (21, 22). This finding is supported by other research indicating that age is a significant predictor of postoperative complications, including pneumonia (23). To mitigate this risk, thorough preoperative assessments should be conducted to identify patients at high risk due to age and comorbidities. Postoperative monitoring should be enhanced, particularly for respiratory function, to detect early signs of pneumonia. Implementing respiratory therapy and physiotherapy can help clear respiratory secretions and improve lung function.

The extent of the surgical procedure is another important factor. More extensive surgeries, such as lobectomy or pneumonectomy, are associated with a higher risk of postoperative pneumonia. This is likely due to the greater tissue disruption and longer operative time, which can lead to increased inflammation and a higher likelihood of respiratory complications. A retrospective study of 956 NSCLC patients found that lobectomy or greater resection was a significant risk factor for postoperative complications, including pneumonia (24). And the extent of lung resection was associated with an increased risk of postoperative pneumonia (25). To reduce this risk, minimally invasive surgical techniques should be used whenever possible to reduce tissue disruption and operative time. Intensive postoperative care, including early mobilization and respiratory support, can reduce the risk of complications (26). Effective pain management strategies should be implemented to facilitate early ambulation and deep breathing exercises (27).

COPD is a well-established independent risk factor for postoperative pneumonia in patients with NSCLC. This conclusion is supported by multiple studies that have investigated the relationship between COPD and postoperative pulmonary complications (28, 29). The prevalence of postoperative pulmonary complications (PPCs) was found to be significantly higher in patients with COPD compared to those with normal lung function (30, 31). Specifically, the incidence of PPCs in patients with COPD was 30.1%, compared to 10.0% in those with normal spirometry (32). This indicates that even in the early stages of COPD, the risk of developing postoperative pneumonia is markedly increased. A more recent study by Takaki Mizoguchi et al (33). retrospectively analyzed 1,139 NSCLC patients who underwent pulmonary resection. The study found that the coexistence of COPD was a significant risk factor for postoperative complications, including atelectasis. This study further supports the notion that COPD significantly increases the risk of postoperative pulmonary complications in NSCLC patients. Given the significant impact of COPD on postoperative pneumonia, several nursing and prevention measures can be implemented to mitigate this risk. Preoperative assessment and optimization are crucial, including thorough preoperative assessments to identify patients with COPD and evaluate their lung function, such as spirometry and arterial blood gas analysis (34). Pulmonary rehabilitation programs can improve respiratory muscle strength and reduce the risk of postoperative complications (35). Postoperative monitoring should be enhanced, particularly for respiratory function, to detect early signs of pneumonia and other pulmonary complications (36).

Smoking history is a well-known risk factor for postoperative pulmonary complications, including pneumonia. Smokers often have impaired respiratory function and a higher prevalence of COPD, which can exacerbate postoperative respiratory issues. However, recent studies have shown that the impact of smoking on postoperative pneumonia has been reduced due to improvements in pre- and postoperative management and surgical techniques (37, 38). Despite this, a history of smoking remains a significant risk factor in many studies, including one that analyzed 232 lung cancer patients with a smoking history. The duration of smoking cessation and the number of pack-years are also important factors to consider, as they can influence the risk of postoperative complications (39, 40). To address this, smoking cessation counseling and support should be provided to patients before surgery. Respiratory rehabilitation programs can improve lung function and reduce the risk of postoperative complications (41). Postoperative monitoring should be intensified for respiratory issues, especially in patients with a history of smoking (42).

Hypoproteinemia is associated with a weakened immune system and reduced wound healing capacity. This can increase the risk of infection and postoperative complications, including pneumonia. Patients with hypoproteinemia may have a higher susceptibility to bacterial infections due to reduced immune function and slower recovery from surgical trauma (43). While hypoproteinemia is not as commonly discussed in the literature as other risk factors, it has been identified in several studies as a significant predictor of postoperative complications in NSCLC patients (44, 45). To manage this risk, nutritional support should be provided to patients with hypoproteinemia to improve their immune function and wound healing. Preoperative nutritional status should be optimized through dietary interventions and supplementation. Postoperative nutritional status should be monitored and managed to prevent further complications (46).

## Conclusion

In conclusion, our study identifies a high incidence rate of postoperative pneumonia, reaching 31.86% within the examined cohort. Through rigorous analysis, several factors—age, extent of the surgical procedure, history of COPD, smoking history, and hypoproteinemia—significantly influence the development of postoperative pneumonia in patients with NSCLC. These factors should be carefully considered in both preoperative assessments and postoperative management protocols to proactively reduce the risk of complications and improve patient outcomes. The predictive model developed in this study demonstrates satisfactory reliability and validity for identifying patients at high risk of postoperative pneumonia. In clinical practice, patients scoring 6 points or higher on this model should be classified as high-risk individuals, warranting heightened vigilance and targeted preventive measures to preempt the onset of postoperative pneumonia. However, external and prospective validation of the proposed model in diverse clinical settings is necessary. Future studies should focus on developing and validating more refined, targeted interventions and preventive strategies to effectively address the identified risk factors. By advancing our understanding and management of these factors, the medical community may work toward reducing the incidence of postoperative pneumonia and enhancing the overall quality of care for NSCLC patients in the postoperative period.

## Data Availability

The original contributions presented in the study are included in the article/supplementary material. Further inquiries can be directed to the corresponding authors.
